# Characterization of a non‐coding RNA‐associated ceRNA network in metastatic lung adenocarcinoma

**DOI:** 10.1111/jcmm.15778

**Published:** 2020-08-29

**Authors:** Feifei Fan, Yu Ping, Li Yang, Xiaoran Duan, Nomathamsanqa Resegofetse Maimela, Bingjie Li, Xiangnan Li, Jing Chen, Kai Zhang, Liping Wang, Shasha Liu, Xuan Zhao, Hongmin Wang, Yi Zhang

**Affiliations:** ^1^ Biotherapy Center The First Affiliated Hospital of Zhengzhou University Zhengzhou China; ^2^ Respiratory and Critical Care Medicine The First Affiliated Hospital of Zhengzhou University Zhengzhou China; ^3^ Cancer Center The First Affiliated of Zhengzhou University Zhengzhou China; ^4^ Department of Thoracic Surgery The First Affiliated Hospital of Zhengzhou University Zhengzhou China; ^5^ School of Life Sciences Zhengzhou University Zhengzhou China; ^6^ Henan Key Laboratory for Tumor Immunology and Biotherapy Zhengzhou China

**Keywords:** ceRNA network, lung adenocarcinoma, metastasis, PPI network, survival

## Abstract

Lung adenocarcinoma (LUAD) is a highly malignant cancer. Although competing endogenous RNA (ceRNA)‐based profiling has been investigated in patients with LUAD, it has not been specifically used to study metastasis in LUAD. We found 130 differentially expressed (DE) lncRNAs, 32 DE miRNAs and 981 DE mRNAs from patients with LUAD in The Cancer Genome Atlas (TCGA) database. We analysed the functions and pathways of 981 DE mRNAs using the Gene Ontology (GO) and the Kyoto Encyclopedia of Genes and Genomes (KEGG) databases. Based on the target DE mRNAs and DE lncRNAs of DE miRNAs, we established an lncRNA‐miRNA‐mRNA ceRNA network, comprising 37 DE lncRNAs, 22 DE miRNAs and 212 DE mRNAs. Subsequently, we constructed a protein‐protein interaction network of DE mRNAs in the ceRNA network. Among all, DE RNAs, 5 DE lncRNAs, 5 DE miRNAs and 45 DE mRNAs were confirmed found to be associated with clinical prognosis. Moreover, 3 DE lncRNAs, 4 DE miRNAs and 9 DE mRNAs in the ceRNA network were associated with clinical prognosis. We further screened 3 DE lncRNAs, 3 DE miRNAs and 3 DE mRNAs using clinical samples. These DE lncRNAs, DE miRNAs and DE mRNAs in ceRNA network may serve as independent biomarkers of LUAD metastasis.

## 1 INTRODUCTION

Lung cancer is one of the most common cancers in the world and has received much attention in recent years due to its association with high morbidity and mortality.^1^ In 2018, the World Health Organization reported the estimated morbidity and mortality associated with lung cancer to be about 11.6% and 18.4%, respectively; in China, its incidence and mortality remain high in both men and women.^2^ Lung cancer has two main subtypes, small‐cell lung cancer (SCLC) and non‐small‐cell lung cancer (NSCLC). Based on pathology, NSCLC has various subtypes, such as adenocarcinoma (LUAD), squamous carcinoma (LUSD) and adenosquamous carcinoma. LUAD, one of the most malignant cancers, is often diagnosed at a late stage and has a poor clinical prognosis.^3^ In addition, it has been reported that LUAD is associated with a high degree of tumour metastasis.^4^, ^5^ Therefore, it is of extreme importance to research the factors associated with lung cancer progression and its poor clinical prognosis.

Cancer progression is closely associated with cancer cell metastasis in LUAD. Using CRISPR/Cas9 technology, Huang *et al* found that catenin knockout attenuates tumour cell metastasis and that catenin effectively enhances cell proliferation and cell cycle through Wnt signalling.^6^ DNA damage results in cell death, although cancer stem cells are able to survive for long periods by avoiding DNA damage. Poly (ADP‐ribose) polymerase 1 promotes DNA repair in cancer cells^7^ and is associated with distant metastasis in patients with LUAD.^8^ High α1‐antitrypsin expression predicts poor prognosis in 88 patients with LUAD and enhances lung cancer cell invasion and metastasis.^9^ The serine protease inhibitor Kazal type 1, a defined prognostic biomarker, was also reported to control cancer cell growth and metastasis.^10^ Although cancer metastasis is regulated by various proteins, it is important to note that there are many other factors that regulate these proteins in the tumour microenvironment.

Long non‐coding RNA (lncRNAs) are a type of non‐coding RNAs that are longer than microRNAs (miRNAs). Numerous recent studies have demonstrated that lncRNAs are abnormally expressed in tumour cells and can interact with miRNA or mRNA to regulate tumour progression. CAR10, a type of lncRNA, is up‐regulated in patients with LUAD and increases cancer cell metastasis and epithelial‐to‐mesenchymal transition by binding to miRNA30 and miRNA203.^11^ MALAT1, HCP5, UCA1 and AS1 have been found to promote tumour development and metastasis, and are associated with the clinical prognosis of LUAD.^12^, ^13^, ^14^, ^15^ MiRNAs, comprising 22 nucleotides, are another type of non‐coding RNAs that regulate mRNA expression by binding to the target region in mRNA.^16^ miRNAs control tumour cell stemness and metastasis by acting either as oncogenes or as tumour suppressors. For example, miRNA‐520c‐3p is expressed at low levels at the tumour site and negatively regulates the biological behaviour of cancer cells through binding to Akt1 and Akt2.^17^ MiRNA‐126‐3p and miRNA‐126‐5p are involved in the progression of LUAD and are positively associated with the TNM stage and metastasis.^18^ In recent years, accumulating evidence has suggested that competing endogenous RNA (ceRNA) competitively binds to miRNA to regulate miRNA or mRNA expression.^19^, ^20^ However, investigation of ceRNAs relationships (either single or multiple) in isolation is not ideal, as the tumour microenvironment is very complex, and isolation studies cannot correctly mimic the complexity of this microenvironment.

The Cancer Genome Atlas (TCGA) is a public database that hosts a huge amount of sequencing data from many types of cancers, including lung, liver, colon and breast.^21^ It is of great importance to study the complex crosstalk within ceRNA networks by analysing sequencing data. Sui *et al* analysed the lncRNA‐miRNA‐mRNA network using TCGA data from patients with LUAD and found many differentially expressed (DE) lncRNAs, DE miRNAs and DE mRNAs in patients with stages I‐II and III‐IV LUAD.^22^ ceRNAs in patients with LUAD have been widely researched in recent years using TCGA data.^23^, ^24^, ^25^ However, ceRNA networks have not been previously investigated in the context of LUAD metastasis. Therefore, our objective was to obtain an in‐depth understanding of the ceRNA network in cancer metastasis, using metastatic and non‐metastatic tumour data from the TCGA database.

## 2 MATERIALS AND METHODS

### 2.1 TCGA data from patients with LUAD

Sequencing data for lncRNA, miRNA and mRNA from patients with LUAD were collected from TCGA (https://portal.gdc.cancer.gov/), and the clinical parameters of all patients were listed (Table S1). lncRNA‐seq and mRNA‐seq data were normalized using the FPKM method according to the formula FPKM = (RC_g_ × 10^9^)/ (RC_pc_ × L). In addition, two separate files (mirnas.quantification.txt and isoforms.quantification.txt) were used to read the 'per million mapped reads' for miRNA‐seq data normalization. Patients with LUAD were screened for further analyses according to the following criteria: 1) LUAD patients with tumours other than lung cancer were excluded; 2) only LUAD patients with clinical information were included; and 3) only LUAD patients with lncRNA, miRNA and mRNA sequencing data were included. We obtained data for 21 LUAD patients with metastasis (M1) and 283 LUAD patients without metastasis (M0).

Forty‐one treatment‐naïve patients with LUAD were enrolled in the First Affiliated Hospital of Zhengzhou University (Zhengzhou, China) and were diagnosed with lung cancer and no other diseases. Ethical approval was obtained from the Human Research Ethics Committee (First Affiliated Hospital of Zhengzhou University, China). Written informed consent was obtained from all participants.

### 2.2 Analysis of DE RNAS (lncRNA, miRNA and mRNA) in M0 and M1 patients

The random variance model t test was used to identify the DE RNAs (lncRNA, miRNA and mRNA) after lncRNA‐seq, mRNA‐seq and miRNA data in M0 and M1 patients were normalized. DE RNAs were screened using the false discovery rate (FDR) method; those with fold change > 1.2 and *P* < .05 were considered significantly different.^26^, ^27^, ^28^ Hierarchical cluster analysis was performed by EPCLUST.

### 2.3 DE mRNA functional enrichment and pathway analysis

DE mRNA functional enrichment was analysed by screening against the Gene Ontology (GO) database to obtain information regarding the biological functions of mRNA.^29^ In addition, Kyoto Encyclopedia of Genes and Genomes (KEGG) analysis was used to enrich the differential pathways in M0 and M1 patients on the basis of the DE mRNAs.^30^ Fisher's exact test was used to select significantly different functions and pathways (*P* < .05).

### 2.4 Construction of cerna network and protein‐protein interaction (PPI) network

On the basis of the 981 DE mRNAs and 32 DE miRNAs, MiRanda (http://www.microrna.org/), Targetscan (http://www.targetscan.org/) and miRWalk (http://129.206.7.150/) were used to screen the target mRNAs of the miRNAs. Next, using miRanda (http://www.microrna.org/) and PITA (https://genie.weizmann.ac.il/pubs/ mir07/mir07_exe.html), we obtained the target lncRNAs of miRNAs based on 32 DE miRNAs and 130 DE lncRNAs. Finally, based on the predicted relationships miRNA‐mRNA and miRNA‐lncRNA, a ceRNA network (lncRNA‐miRNA‐mRNA) was constructed.^31^

We selected the DE mRNAs in the ceRNA network and predicted the protein‐protein interactions (PPIs) using the String database. Finally, a PPI regulatory network was constructed using Cytoscape 3.1.0 (http://cytoscape.org/).^32^, ^33^, ^34^ The protein interaction score was > 0.4 in this PPI network.

### 2.5 Survival analysis of DE lncRNAs, DE miRNAs and DE mRNAs

Patients with LUAD and survival information in the TCGA database were divided into two groups, low expression and high expression, according to the medians of DE lncRNAs, DE miRNAs and DE mRNAs, respectively. Then, these DE RNAs were selected to analyse survival using the univariate Cox proportional hazards regression model. However, this method was not applicable to analyse the survival curves crossed, and therefore, the two‐stage procedure was used. *P* < .05 was considered statistically significant.

### 2.6 RNA extraction

Tumour and normal tissues from the 41 patients with LUAD were collected for RNA extraction. Tissues were cut into small pieces and placed into RNase‐free tubes, and RNA was extracted using Trizol (Takara). Briefly, chloroform was used to separate RNA, which was precipitated with isopropanol. Finally, RNA was washed in 75% ethanol and the RNA concentration was measured using Nanodrop 2000 (Agilent).

### 2.7 Real‐time quantitative polymerase chain reaction (QRT‐PCR)

cDNA was synthesized from mRNA and lncRNA according to the manufacturer´s instructions (Takara, Japan), and the miScript II RT kit (Qiagen) was used for cDNA synthesis from miRNA. Finally, the SYBR Premix Ex Taq was used for qRT‐PCR according to the manufacturer's instructions using the following reaction conditions: 95°C for 30 s, followed by 40 cycles of 94°C for 5 s and 60°C for 30 s. Each 20 μL reaction included 10 μL SYBR Green (Roche), 2 μL cDNA which was diluted 5‐10 times, 10 μ M primer forward 0.8 μL, 10 μM primer reverse 0.8 μL and 6.4 μL water.

### 2.8 Statistical analysis

GraphPad Prism 7.00 was used to construct histograms. All data in qRT‐PCR were expressed as the mean ± the standard error of the mean (SEM). *P* < .05 was statistically significant. Student's *t* test was used to compare the qRT‐PCR results. DE RNAs in TCGA were identified by the random variance model *t* test. Log‐rank test and two‐stage procedure were used for Kaplan‐Meier survival curve analysis. Cox regression analysis was used to assess the relationship between DE RNAs and survival time.

## 3 RESULTS

### 3.1 DE RNAs (lncRNA, miRNA and mRNA) in patients with LUAD

**Figure 1:**
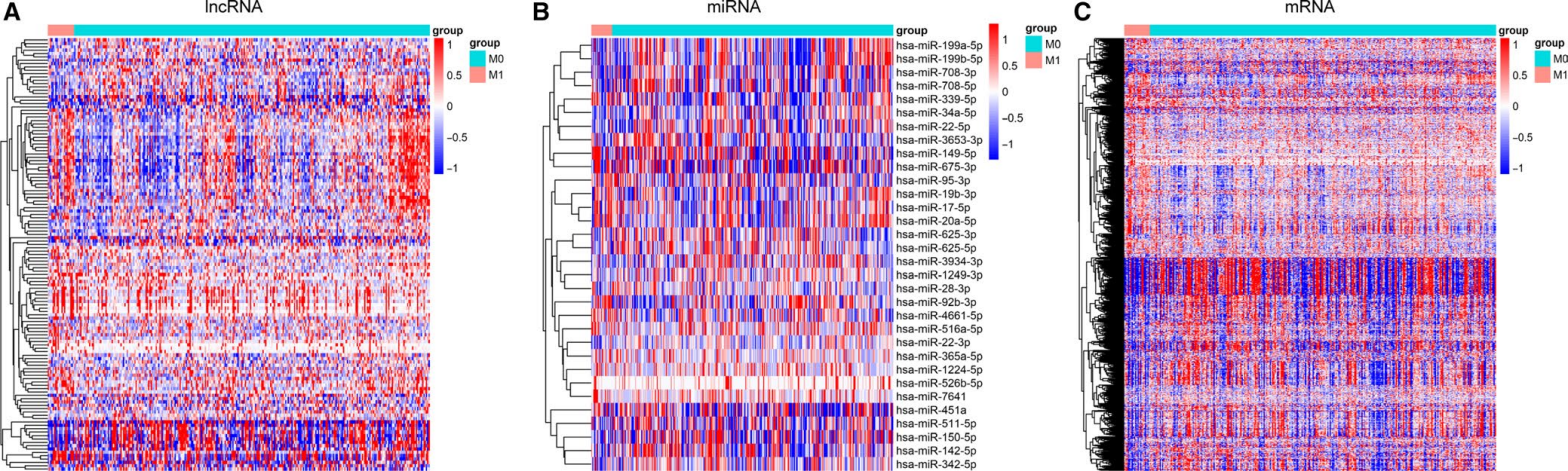
Hierarchical clustering dendrograms of patients with lung adenocarcinoma. The heat map reflects 130 differentially expressed (DE) lncRNAs (A), 32 DE miRNAs (B), and 981 DE mRNAs (C). Each column represents a patient and each row represents miRNA, lncRNA or mRNA. Red colour represents up‐regulation, and blue colour represents down‐regulation

Patients with LUAD (n = 304) from the TCGA database were classified into two groups, M0 (n = 283) and M1 (n = 21); data from these patients were screened for metastasis‐associated factors (Figure S1). First, we compared these two groups and obtained the DE RNAs, 130 lncRNAs, 32 miRNAs and 981 mRNAs (*P* < .05 and fold change > 1.2; Table S2, Figure 1). The top 5 up‐regulated lncRNAs were H19, RP11‐284F21.10, ERVH48‐1, CTD‐2139B15.5 and RP11‐284F21.7. The top 5 down‐regulated lncRNAs were RNU4‐62P, RP11‐379F4.6, RP11‐1334A24.6, SNORA2 and AC009237.8 (Table 1). The top 5 up‐regulated miRNAs were hsa‐miR‐675‐3p, hsa‐miR‐4661‐5p, hsa‐miR‐1224‐5p, hsa‐miR‐149‐5p and hsa‐miR‐451a. The top 5 down‐regulated miRNAs were hsa‐miR‐28‐3p, hsa‐miR‐1249‐3p, hsa‐miR‐22‐3p, hsa‐miR‐625‐3p and hsa‐miR‐342‐5p (Table 2). The top 5 up‐regulated mRNAs were ALDH3A1, INHA, UCN3, SCG2 and VGF. The top 5 down‐regulated mRNAs were LSM10, NLRP3, TWF2, PRICKLE2 and MGAT1 (Table 3).

**Table 1:** Top 5 up‐regulated and down‐regulated long non‐coding RNAs

Ensembl_Gene_ID	Gene Symbol	Adjusted *P*‐value	Regulation	Fold change
ENSG00000130600	H19	.0178877	Up	2.5
ENSG00000272405	RP11‐284F21.10	.0064564	Up	1.99
ENSG00000233056	ERVH48‐1	.0002666	UP	1.95
ENSG00000249199	CTD‐2139B15.5	.0019603	Up	1.85
ENSG00000229953	RP11‐284F21.7	.006571	UP	1.75
ENSG00000222057	RNU4‐62P	.0410318	Down	0.83
ENSG00000272440	RP11‐379F4.6	.0462578	Down	0.83
ENSG00000248996	RP11‐1334A24.6	.0297649	Down	0.83
ENSG00000202343	SNORA2	.0491674	Down	0.83
ENSG00000229689	AC009237.8	.0355755	Down	0.82

**Table 2:** Top 5 up‐regulated and down‐regulated micro (mi)RNAs

miRNA	Adjusted *P*‐value	Regulation	Fold change
hsa‐miR‐675‐3p	.0409407	Up	2.14
hsa‐miR‐4661‐5p	.0007762	Up	1.83
hsa‐miR‐1224‐5p	.0001262	Up	1.76
hsa‐miR‐149‐5p	.0291066	Up	1.66
hsa‐miR‐451a	.0388346	Up	1.61
hsa‐miR‐28‐3p	.0458777	Down	0.83
hsa‐miR‐1249‐3p	.0377803	Down	0.82
hsa‐miR‐22‐3p	.007913	Down	0.77
hsa‐miR‐625‐3p	.0287589	Down	0.74
hsa‐miR‐342‐5p	.0086302	Down	0.73

**Table 3:** Top 5 up‐regulated and down‐regulated mRNAs

Ensembl_Gene_ID	Gene Symbol	Adjusted *P*‐value	Regulation	Fold change
ENSG00000108602	ALDH3A1	.0192206	Up	2.39
ENSG00000123999	INHA	.0049966	Up	2.24
ENSG00000178473	UCN3	.00329	Up	2.16
ENSG00000171951	SCG2	.0008007	Up	2.05
ENSG00000128564	VGF	.0000141	Up	2
ENSG00000198951	NAGA	.0138281	Down	0.83
ENSG00000183597	TANGO2	.0137221	Down	0.83
ENSG00000174917	C19orf70	.0494865	Down	0.83
ENSG00000011132	APBA3	.0140741	Down	0.83
ENSG00000186111	PIP5K1C	.0254345	Down	0.83

### 3.2 Go enrichment and KEGG pathways associated with de mRNAmRNA

**Figure 2:**
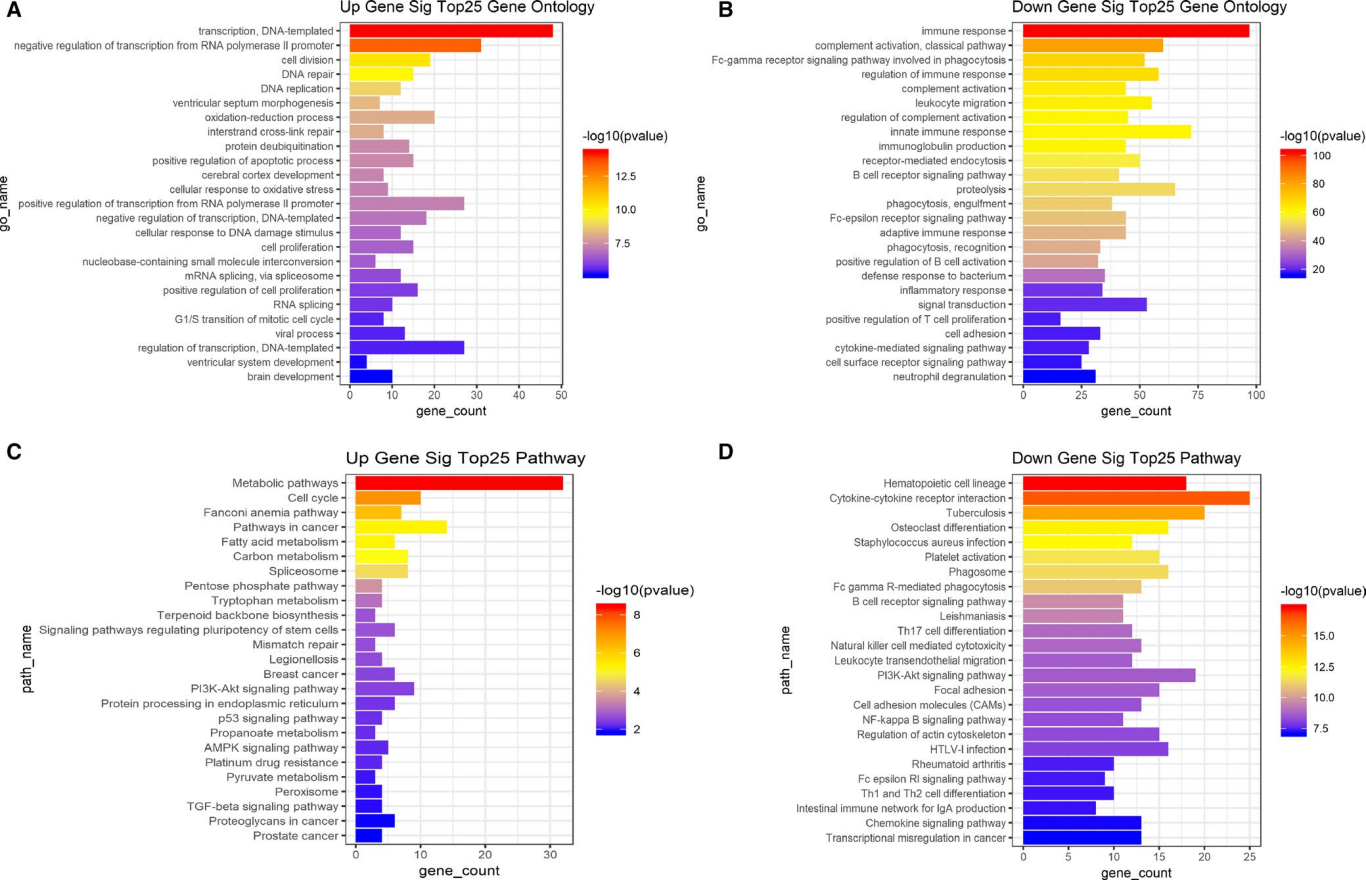
GO enrichment and KEGG pathways of differentially expressed mRNA in patients with lung adenocarcinoma. Panels A and B show the top 25 GO functions with the most significant *P*‐values for the up‐ and down‐regulated mRNAs, respectively. The x‐axis represents the number of mRNAs involved in the GO function. Panels C and D show the top 25 KEGG pathways with the most significant *P*‐values of the up‐ and down‐regulated mRNAs, respectively. The x‐axis represents the number of mRNAs involved in the KEGG pathway

In patients with LUAD, 981 DE mRNAs were annotated according to information in the GO database. We enriched the functions of these mRNAs (*P* < .05) using GO. The top 25 GO functions of the up‐regulated mRNAs are listed in Figure 2A, whereas the top 25 GO functions of the down‐regulated mRNAs are listed in Figure 2B. Among these GO‐enriched functions, cell division, DNA repair and cell proliferation are positively associated with cell growth and metastasis.

To further identify the biological processes associated with the 981 DE mRNAs, we analysed the KEGG pathways associated with these mRNAs. We found that the up‐regulated mRNAs were positively associated with some KEGG pathways, such as the metabolic pathway, cell cycle, p53 and PI3K‐Akt signalling pathways, and signalling pathways regulating stem cell pluripotency (Figure 2C). These pathways have all been reported to promote cancer progression and metastasis.^35^, ^36^ Meanwhile, down‐regulated mRNAs were associated with the B cell receptors, cell adhesion molecules and chemokine signalling pathways (Figure 2D).

### 3.3 Cerna regulatory network in patients with LUAD

**Figure 3:**
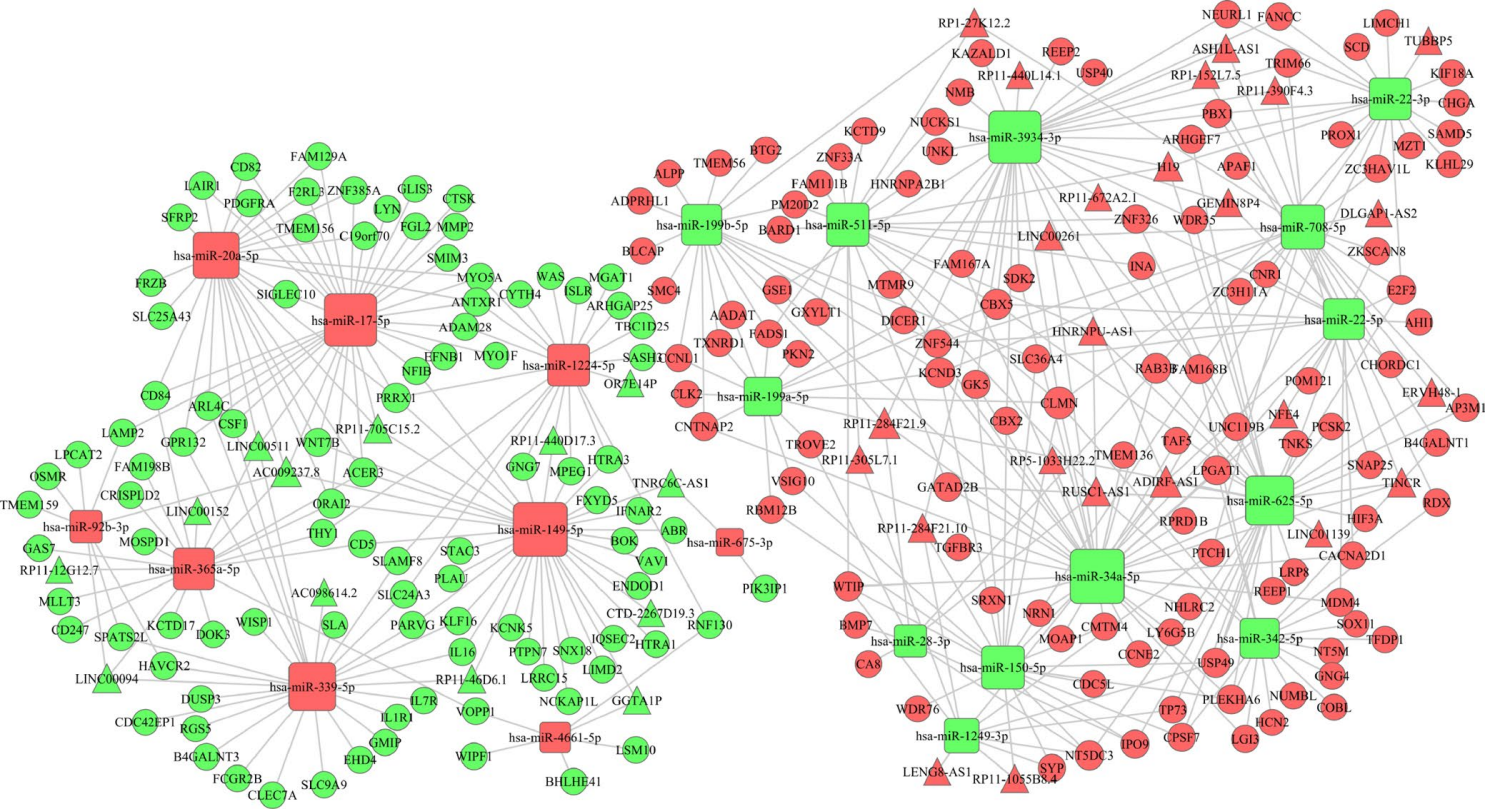
The lncRNA‐miRNA‐mRNA ceRNA network in patients with LUAD. The nodes highlighted in red indicate up‐regulation, whereas the nodes highlighted in blue indicate down‐regulation in patients with metastasis. Triangles, rectangles and circles represent lncRNAs, miRNAs and mRNAs, respectively. The bigger nodes represent stronger regulation ability, and the smaller nodes represent weaker regulation ability

Next, using the miRanda, Targetscan and miRWalk websites, we predicted the target mRNAs of 32 DE miRNAs from among the 981 mRNAs screened. Then, using the miRanda and PITA websites, we selected the target lncRNAs of the 32 DE miRNAs from among the 130 DE lncRNAs. By combining the aforementioned data, the lncRNA‐miRNA‐mRNA ceRNA network was constructed through screening the miRNA‐mRNA and miRNA‐lncRNA targeting (Figure 3). Finally, we obtained 22 dominant nodes of miRNAs (9 up‐regulated and 13 down‐regulated miRNAs). In this ceRNA network, some of the miRNAs (such as miRNA625, miRNA20a and miRNA149) have been reported to serve as tumour suppressors or oncogenes.

### 3.4 PPI regulatory network in patients with LUAD

**Figure 4:**
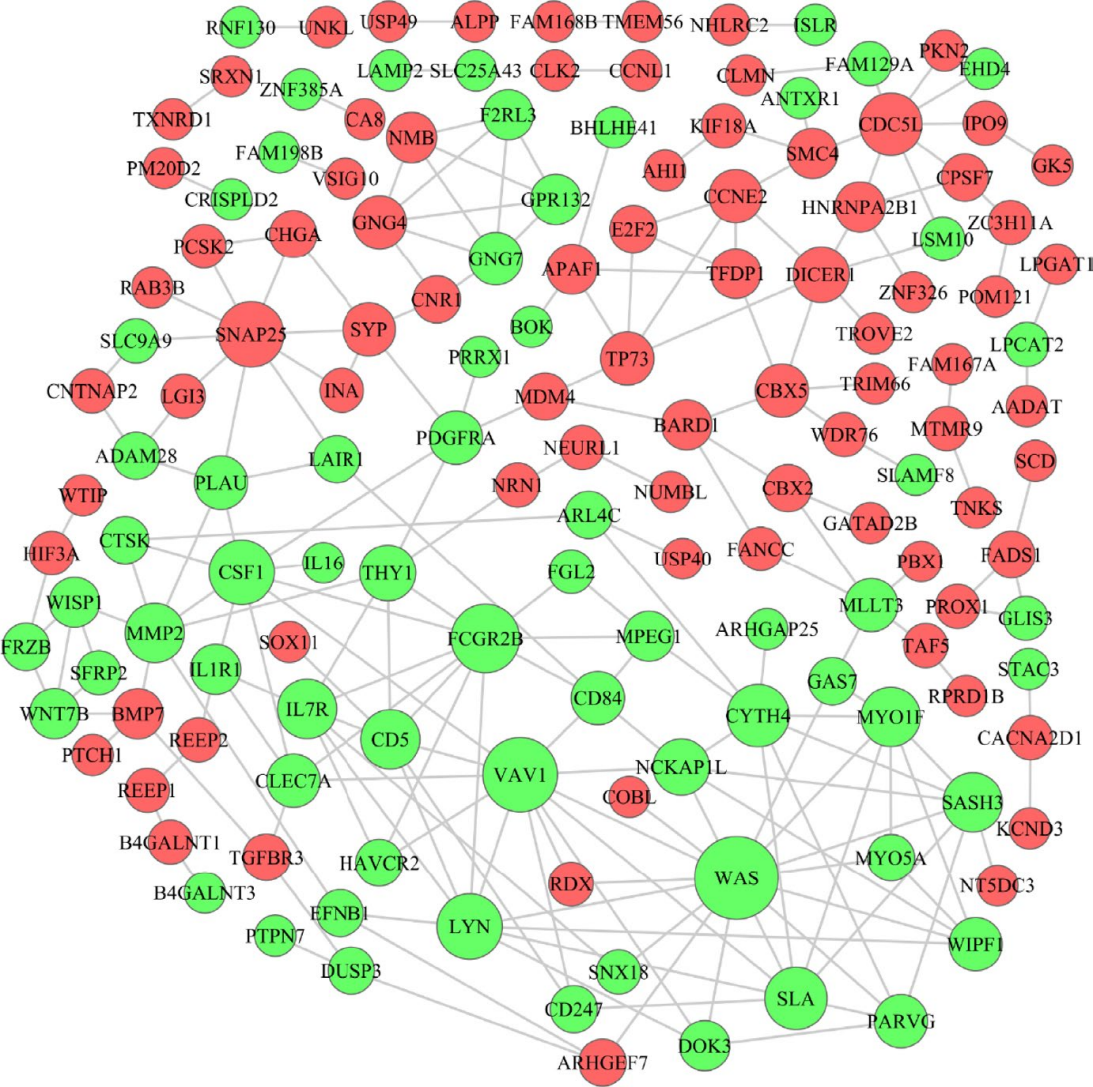
The protein‐protein interaction (PPI) regulatory network in patients with LUAD. The nodes highlighted in red indicate up‐regulation, whereas the nodes highlighted in blue indicate down‐regulation in patients with metastasis. The bigger nodes represent stronger regulation ability, and the smaller nodes represent weaker regulation ability

Based on the mRNAs in the lncRNA‐miRNA‐mRNA ceRNA network, we established a PPI regulatory network using the String database to better present the protein‐protein interaction in M1 patients with LUAD (Figure 4). In the PPI regulatory network, the proteins, SNAP25, CCNE2, CDC5L and DICER1, were up‐regulated and had a strong regulatory capacity; FCGR2B, VAV1, WAS, LYN and CSF1 were down‐regulated proteins that play important roles.

### 3.5 Association between survival and the expression of lncRNAs, miRNAs and mRNAs in the cerna network in patients with LUAD

**Figure 5:**
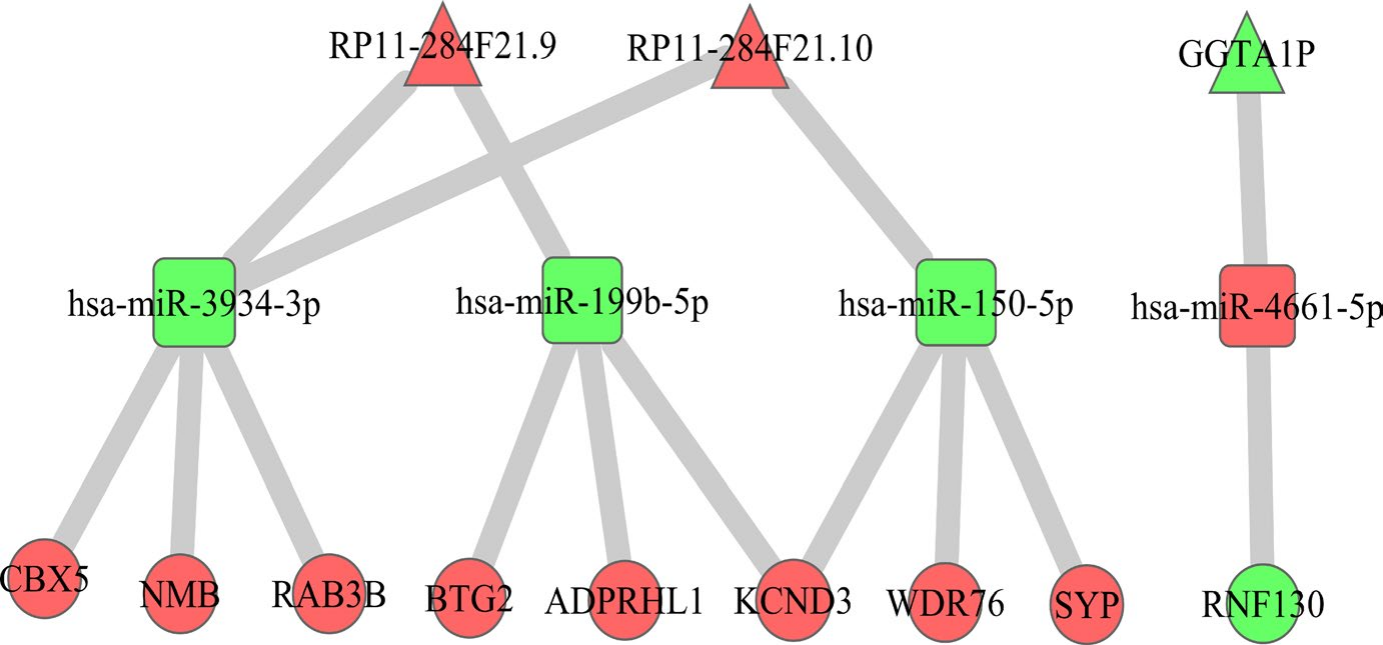
Mutual regulatory relations among 9 survival‐associated differentially expressed (DE) mRNAs, 3 DE lncRNAs and 4 DE miRNAs. The nodes highlighted in red indicate up‐regulation, whereas the nodes highlighted in blue indicate down‐regulation in patients with metastasis. Triangles, rectangles and circles represent lncRNAs, miRNAs and mRNAs, respectively

**Figure 6:**
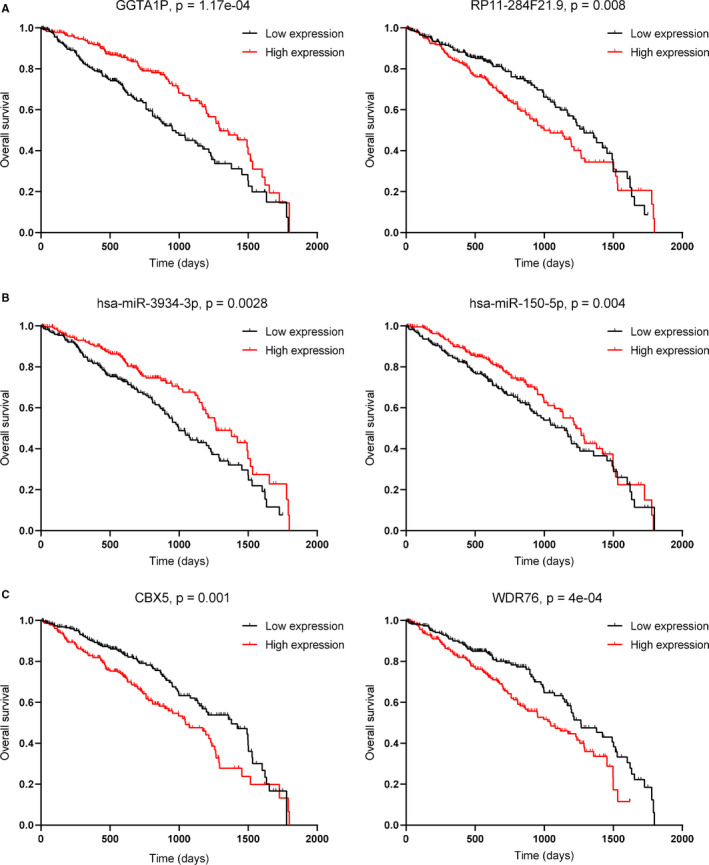
Kaplan–Meier curve for lncRNAs (A), miRNAs (B) and mRNAs (C). The x‐axis represents time (days), and the y‐axis represents the overall survival

To further explore whether metastasis‐associated lncRNAs, miRNAs and mRNAs in the ceRNA network were associated with clinical prognosis, the Kaplan‐Meier survival curves for these molecules were generated. Finally, 5 DE lncRNAs, 5 DE miRNAs and 45 DE mRNAs were associated with survival (*P* < .05). Moreover, 3 DE lncRNAs, 4 DE miRNAs and 9 DE mRNAs in the ceRNA regulatory network were associated with clinical prognosis and were able to regulate each other (Figure 5). Patients with higher expression of GGTA1P had better prognosis than those with a lower expression (Figure 6A). In contrast, patients with lower expression of RP11‐284F21.9 showed a better clinical prognosis than those with higher expression (Figure 6A). In patients with LUAD, low expression of hsa‐miR‐3934‐3p and hsa‐miR‐150‐5p was correlated with poor prognosis (Figure 6B). CBX5 and WDR76 were highly expressed in M1 patients, and survival analysis showed that patients expressing low CBX5 and WDR76 had better clinical prognosis (Figure 6C). In addition, we performed uni‐factor cox regression analysis to examine the relationship between these DE RNAs and prognostic of lung cancer patients (Figure S2). We found that five of the six DE RNAs could be independent prognosis factors to predict prognosis of lung cancer patients. Especially, GGTA1P, RP11‐284F21.9, hsa‐miR‐150‐5p and CBX5 showed statistical significance as independent prognostic factors, whereas these RNAs had crossover in KM survival analysis to separate lung cancer patients’ survival outcomes. Hsa‐miR‐3934‐3p could not be an independent prognostic factor in this analysis, which was possibly due to the small amount of miRNA samples with clinical data in TCGA. Overall, the results of cox regression analysis were consistent with the results of KM survival curve.

### 3.6 Expression of 3 DE lncRNAs, 4 DE miRNAs and 9 DE mRNAs in 41 patients with LUAD

**Figure 7:**
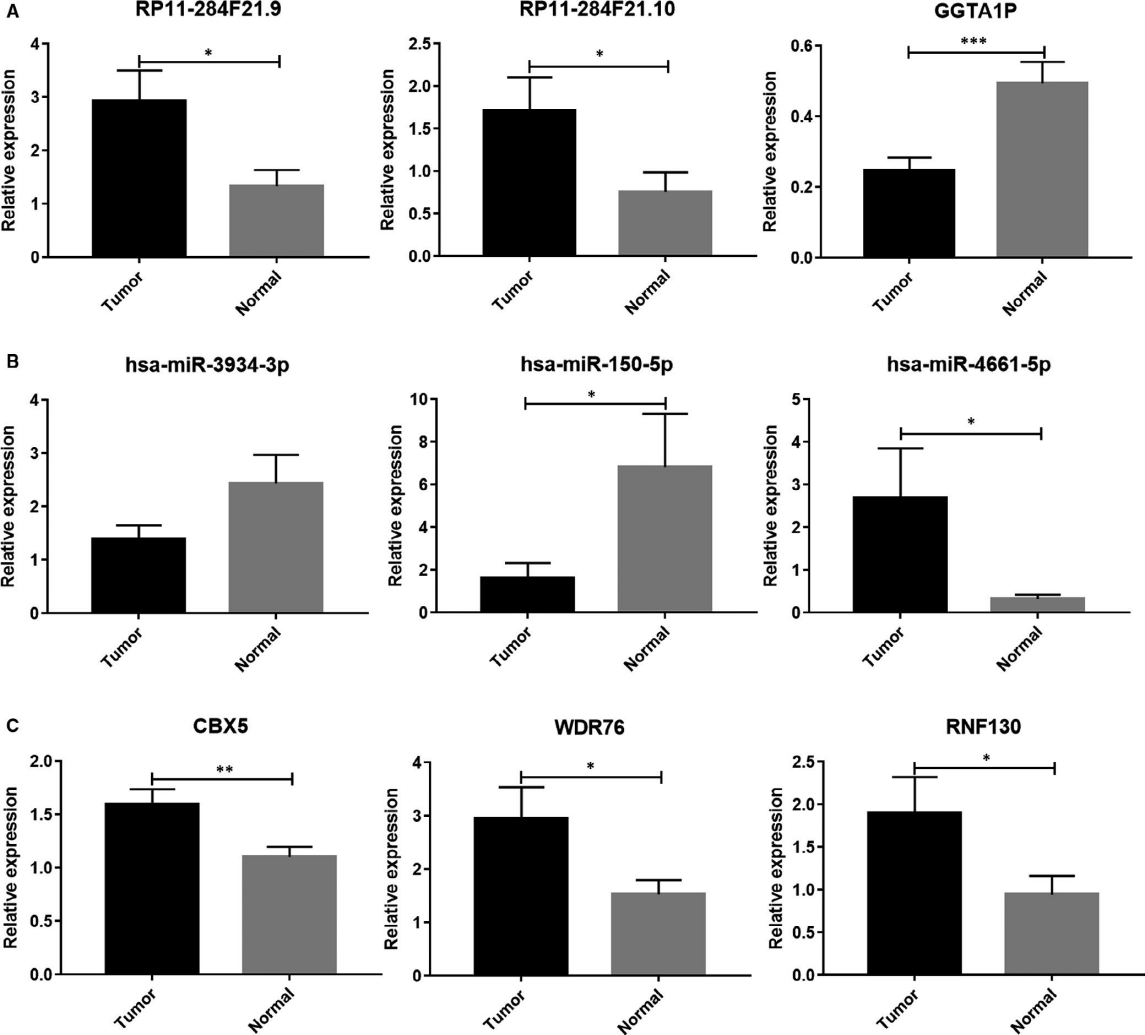
Expression of differentially expressed (DE) lncRNAs (A), DE miRNAs (B) and DE mRNAs (C) in tumour and normal tissues. Tumour and normal tissues from 41 patients with LUAD were used to extract RNA and perform RT‐PCR for DE RNA detection

After screening the DE RNAs (3 DE lncRNAs, 4 DE miRNAs and 9 DE mRNAs) in the ceRNA regulatory network, we further investigated the expression of these DE RNAs through the Gene Expression Profiling Interactive Analysis (GEPIA) server and using qRT‐PCR, to enhance the reliability of our bioinformatic results. Data from 304 patients in the TCGA database were used for screening these DE RNAs in the results already reported here. Next, we reanalysed the expression of 3 DE lncRNAs and 9 DE mRNAs in 483 tumour tissue and 59 normal tissue samples from the TCGA database using GEPIA. It was confirmed that the expression of 3 DE lncRNAs, 3 DE mRNAs—regulated by hsa‐miR‐3934‐3p—1 DE mRNA—regulated by hsa‐miR‐199b‐5p—1 DE mRNA—regulated by hsa‐miR‐150‐5p—and 1 DE mRNA—regulated by hsa‐miR‐4661‐5p—in the tumour tissue exhibited trends similar to that observed in the ceRNA regulatory network (Figure S3). In addition, the expression of 4 DE miRNAs was investigated by qRT‐PCR, and the expression of hsa‐miR‐3934‐3p, hsa‐miR‐150‐5p and hsa‐miR‐4661‐5p was consistent with the results in the ceRNA regulatory network (Figure 7B). Therefore, based on the aforementioned results, 3 DE lncRNAs and 5 DE mRNAs (CBX5, NMB, RAB3B, WDR76 and RNF130) were further analysed using qRT‐PCR. The expression of 3 DE lncRNAs (RP11‐284F21.9, RP11‐284F21.10 and GGTA1P) and 3 DE mRNAs (CBX5, WDR76 and RNF130) in the 41 samples was similar to that of the results of bioinformatic analyses, whereas 2 DE mRNAs NMB and RAB3B showed no difference in expression between tumour and normal tissue (Figure 7A and C). Finally, the 41 patients with LUAD were stratified into two groups, M0 and M1. Using qRT‐PCR, we were able to verify that 3 lncRNAs, 3 miRNAs and 3 mRNAs exhibited similar expression trends as those predicted using bioinformatic analyses (Figure S4).

## 4 DISCUSSION

In this study, we aimed to investigate the factors affecting metastasis in patients with LUAD. To comprehensively analyse these factors, we obtained RNA sequencing data (lncRNA, miRNA and mRNA) from the TCGA database. We used bioinformatics to predict and explore cancer development and metastasis. First, we screened 981 DE mRNAs, 130 DE lncRNAs and 32 DE miRNAs, and found that they were dysregulated in M0 but not in M1 patients with LUAD. Then, we further analysed the functions of these differentially expressed mRNAs based on the GO and KEGG pathway databases. We established a ceRNA network, based on the DE lncRNAs, DE miRNAs and DE mRNAs, to visualize the interactions among these RNAs. In addition, we analysed the association between the differentially expressed RNAs in the ceRNA network and clinical prognosis in patients with LUAD. Therefore, this study helps to provide a better understanding of the mechanisms underlying cancer metastasis.

MiRNAs have been reported to control the biological behaviour of tumour cells via regulating mRNA expression.^37^, ^38^, ^39^ In our study, we first predicted that 32 DE miRNAs were associated with metastasis. Hsa‐miR‐675 is known to regulate the hypoxia‐associated epithelial‐to‐mesenchymal transition in colon cancer cells, suggesting that hsa‐miR‐675 is associated with tumour cell stemness and metastasis.^40^ It has been reported that the down‐regulation of hsa‐miR‐149 expression is associated with cancer stem cell apoptosis in prostate cancer.^41^ Here, we found that the expression of hsa‐miR‐675 and hsa‐miR‐149 was higher in M1 patients than in M0 patients, which was consistent with the findings of previous studies. With the exception of hsa‐miR‐675 and hsa‐miR‐149, other miRNAs associated with metastasis such as hsa‐miR‐28, hsa‐miR‐22 and hsa‐miR‐150 were identified. Hsa‐miR‐22 has been reported to inhibit tumour cell migration and invasion in colon cancer.^42^ It has been shown that high expression of hsa‐miR‐28 is associated with poor overall survival and relapse in patients with colorectal adenocarcinoma.^43^ The findings of these studies are consistent with our results indicating that DE miRNAs were able to mediate cancer cell progression and metastasis.

In recent years, lncRNAs have become a popular topic in cancer research and have been widely and thoroughly studied in various cancers.^44^ In our study, we found variation in the expression levels of lncRNAs (including H19, RP11‐284F21.10, RP11‐1334A24.6 and SNORA2) in M1 patients with LUAD. LncRNAs usually perform their biological functions by competitively binding to miRNAs. For example, H19 was able to protect cells from hypoxia‐induced injury by competitively binding to hsa‐miR‐139.^45^ Wang *et al* demonstrated that the interaction of H19 and hsa‐miR‐675‐5p mediates breast cancer progression.^46^ In our study, we constructed the lncRNA‐miRNA‐mRNA ceRNA network of patients with metastasis to further the understanding of how non‐coding RNA regulates mRNA expression. Xu *et al* used this bioinformatics method to establish a ceRNA network for 20 types of cancers, such as ovarian and lung cancer, and glioma.^47^ Reportedly, ceRNA networks can also be constructed to analyse the regulatory factors in one signalling pathway.^48^

Here, we found that the DE lncRNAs (GGTA1P, LINC00125, LINC00261, RP11‐284F21.9 and RP11‐284F21.1) in the ceRNA network were negatively or positively associated with clinical prognosis in patients with LUAD. In triple‐negative breast cancer, patients with higher GGTA1P expression exhibit better relapse‐free survival.^49^ LINC00261 expression plays an important role in cell proliferation, apoptosis and invasion in choriocarcinoma and gastric cancer,^50^, ^51^ and was identified as a novel prognostic biomarker in pancreatic cancer.^52^ Additionally, LINC00261 is able to bind to hsa‐miR‐182, hsa‐miR‐183, hsa‐miR‐153 and hsa‐miR‐27a; further, hsa‐miR‐96 was able to regulate FOXO1 mRNA expression, suppressing cell proliferation, migration and invasion of endometrial cancer cells.^53^ Finally, based on the survival analysis of DE RNAs in ceRNA network and experimental data, we identified 3 lncRNAs, 3 miRNAs and 3 mRNAs. We will further investigate which mRNAs play major roles in regulating metastasis, and which lncRNAs and miRNAs regulate these mRNAs.

In conclusion, we used an integrative biological approach to analyse DE mRNAs, lncRNAs and miRNAs in patients with LUAD in the M0 and M1 patient groups. Moreover, we obtained information from the GO and KEGG databases to understand the functions and pathways associated with these screened mRNAs, lncRNAs and miRNAs. The lncRNA‐miRNA‐mRNA ceRNA network and PPI networks were established, revealing a novel regulatory mechanism for further investigation of LUAD. Finally, we also screened the DE mRNAs, lncRNAs and miRNAs in the network with respect to the clinical prognosis (using RT‐PCR), which may serve as independent biomarkers of LUAD metastasis.

## CONFLICT OF INTEREST

The authors confirm that there are no conflicts of interest.

## **AUTHOR** **CONTRIBUTIONS**

FFF, HMW and YZ: Study design. YP, LY, XRD and NRM: Data analysis. BJL, XNL, JC, KZ and LPW: Sample collection. SSL and XZ: Data analysis. All authors read and approved the final manuscript.

## Supporting information

Fig S1

Fig S2

Fig S3

Fig S4

Table S1

Table S2

## Data Availability

The data that support the findings of this study are available from the corresponding author upon reasonable request.
