# Supplementary figures and images for: Characterization of a non‐coding RNA‐associated ceRNA network in metastatic lung adenocarcinoma
Source: J Cell Mol Med. 2020 Aug 29;24(20):11680–90. doi: 10.1111/jcmm.15778 (PMC7579711; doi:10.1111/jcmm.15778)

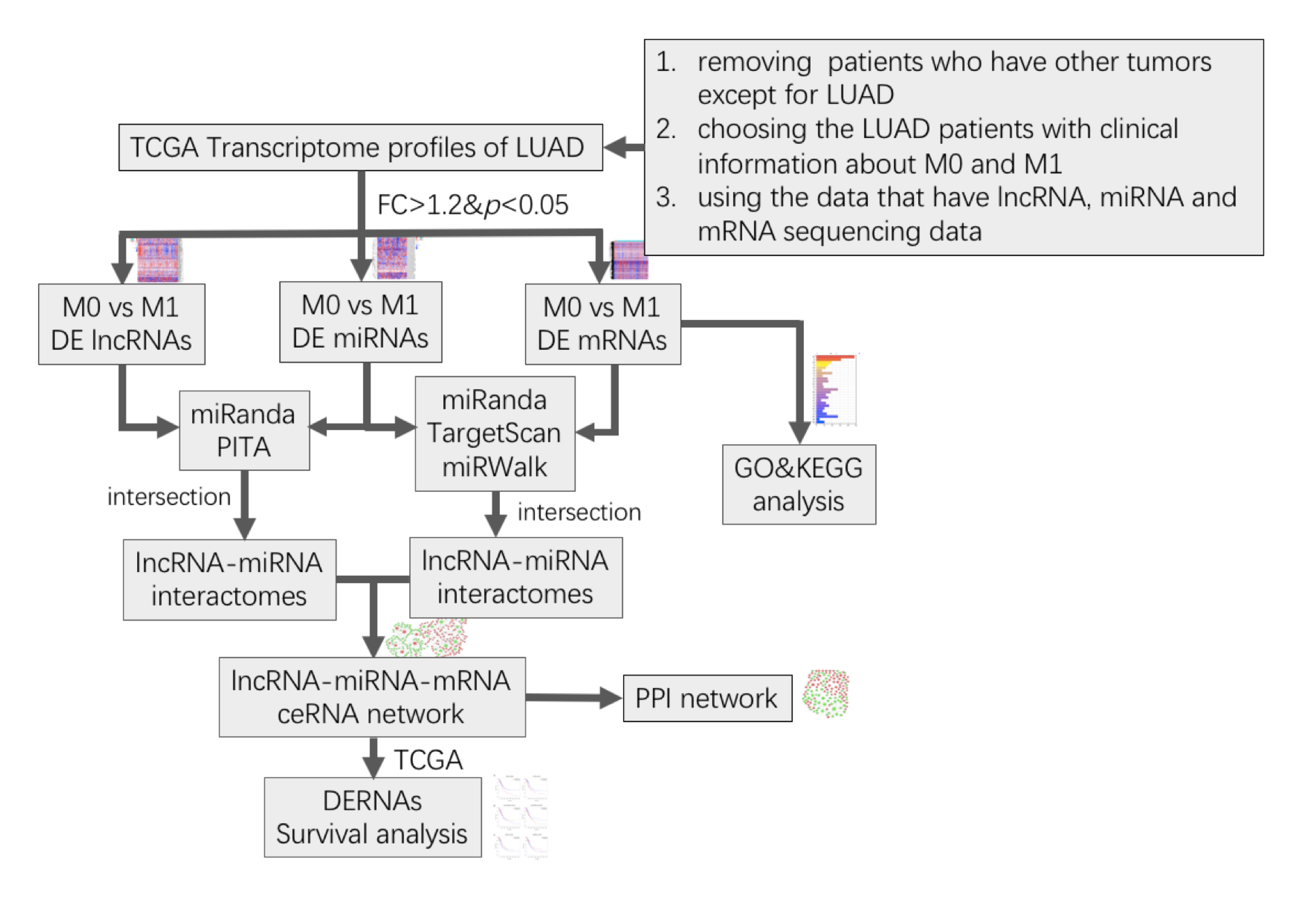

Supplement: Supplementary file 1 — Fig S1 [file JCMM-24-11680-s001.tif]

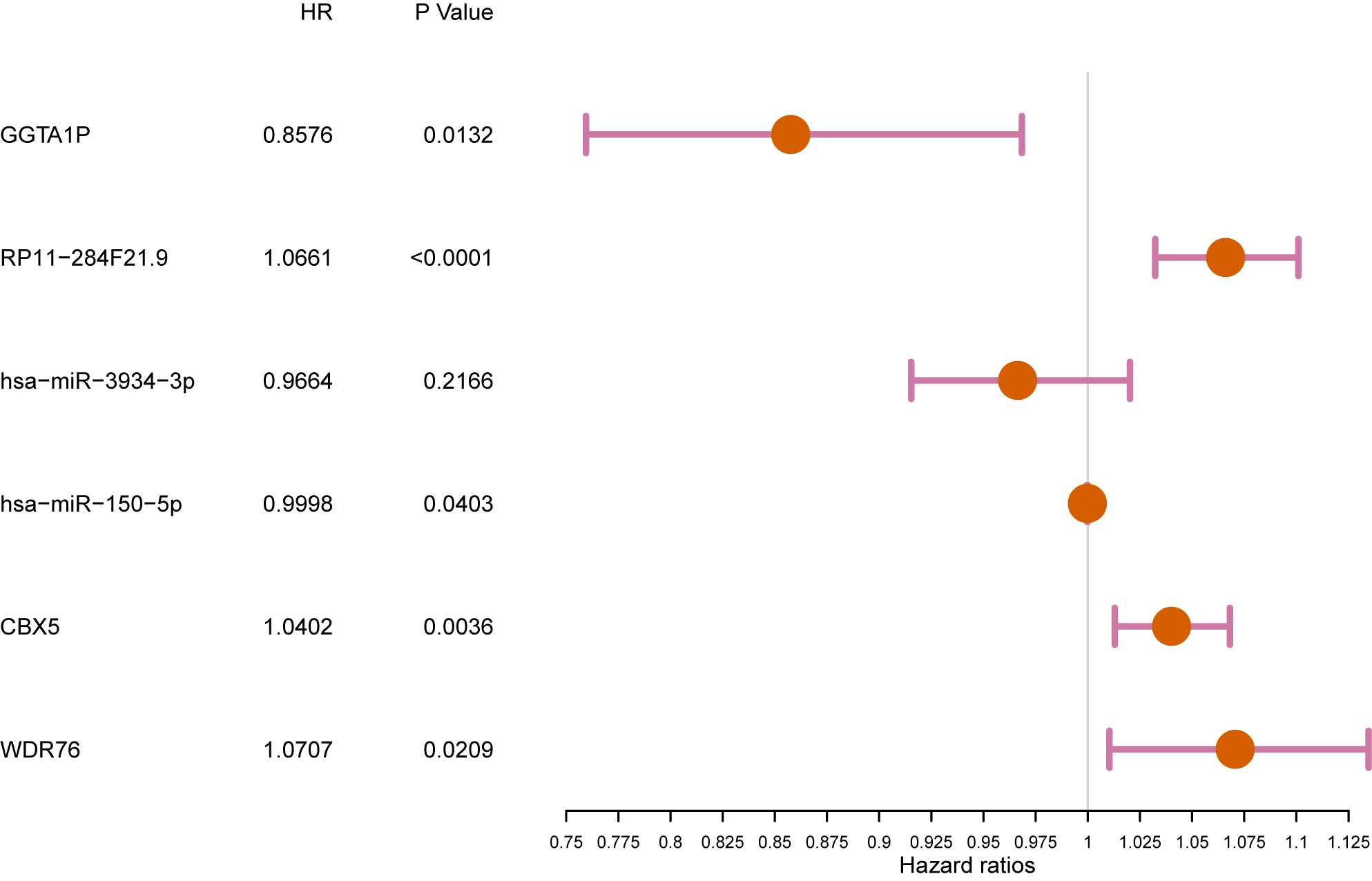

Supplement: Supplementary file 2 — Fig S2 [file JCMM-24-11680-s002.tif]

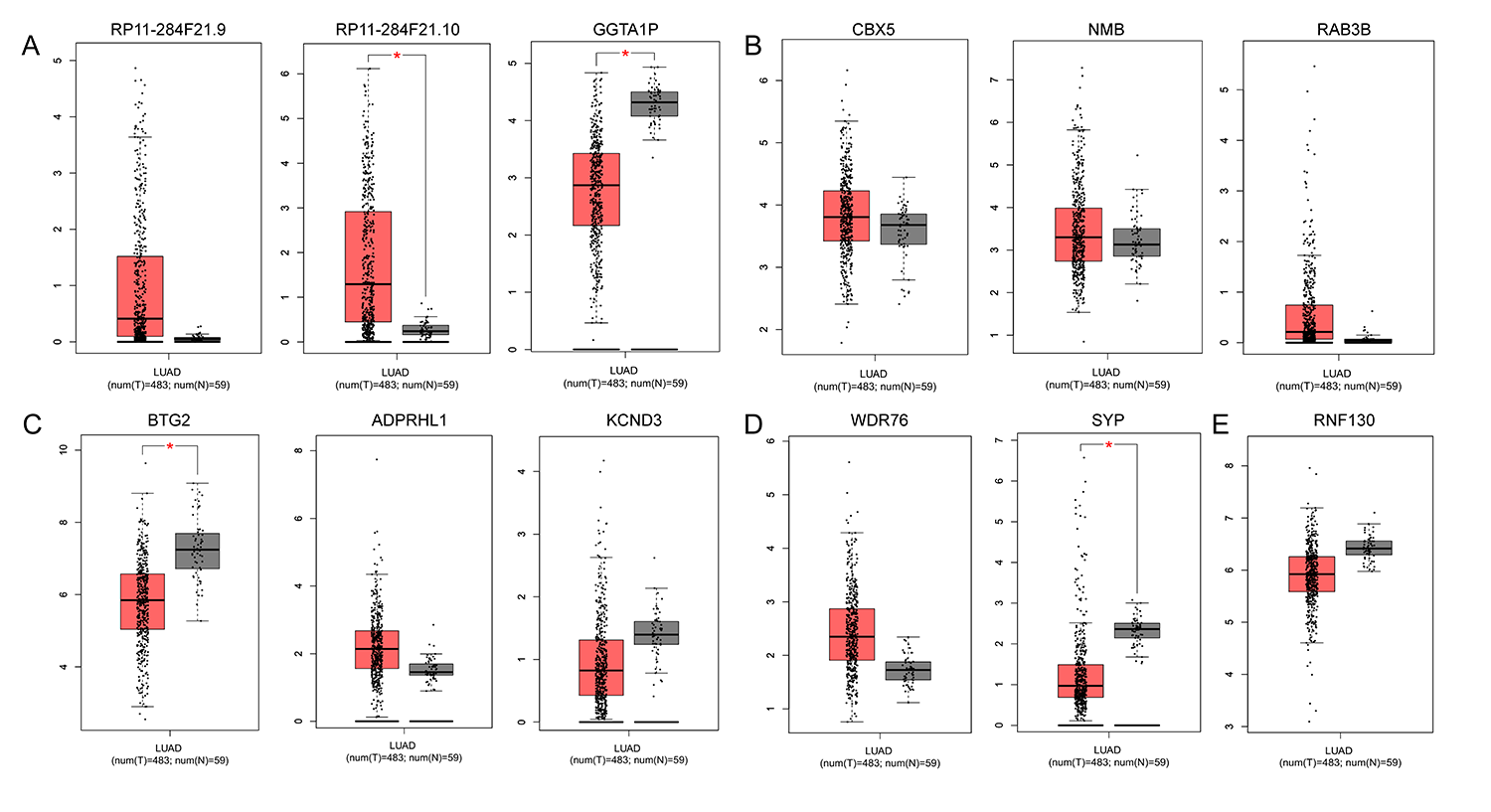

Supplement: Supplementary file 3 — Fig S3 [file JCMM-24-11680-s003.tif]

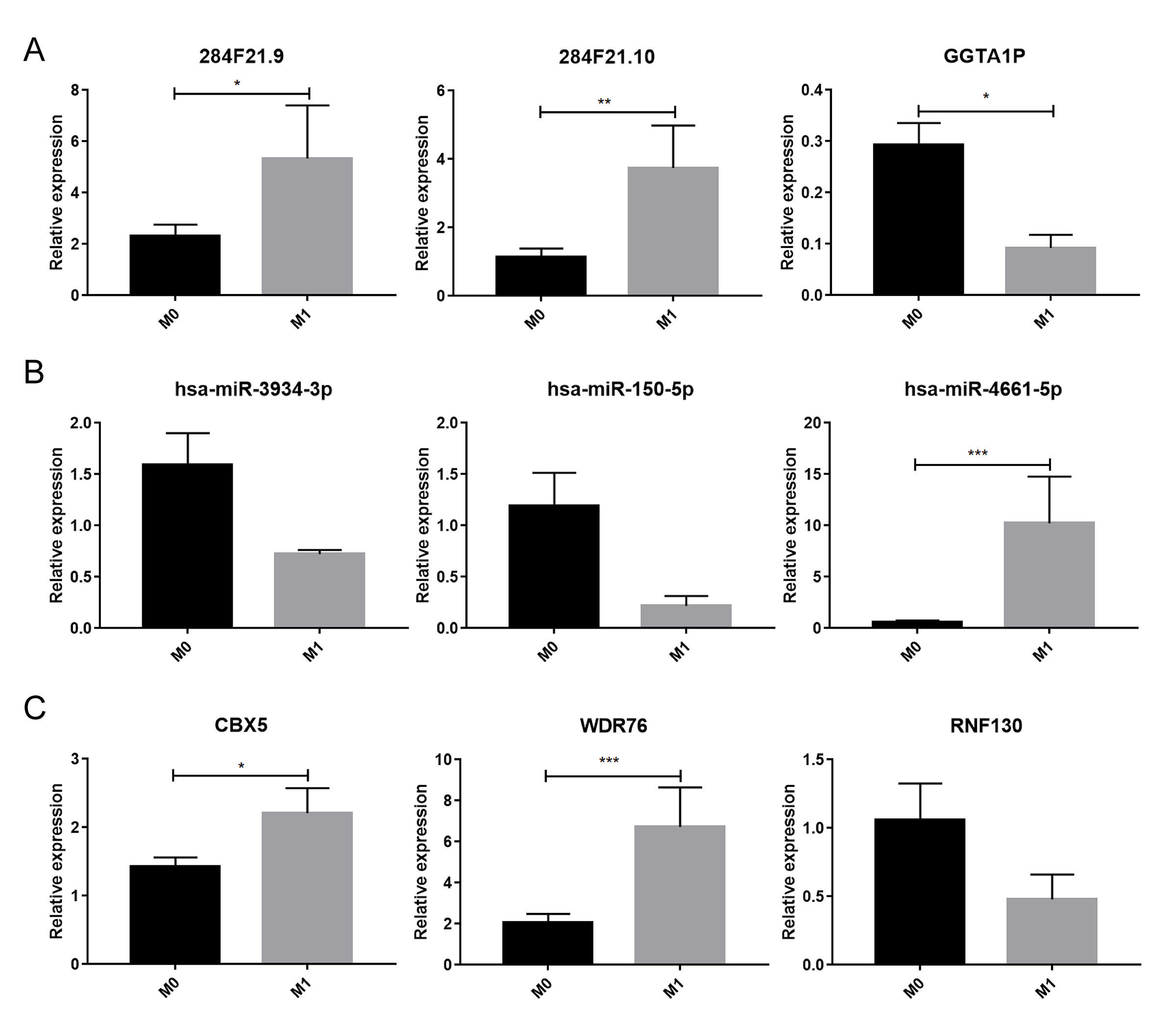

Supplement: Supplementary file 4 — Fig S4 [file JCMM-24-11680-s004.tif]
